# Supplementary material for: Large private shareholders, industrial policies and industrial loans of city commercial banks: Evidence from China
Source: PLoS One. 2022 Dec 8;17(12):e0278654. doi: 10.1371/journal.pone.0278654 (PMC9731413; doi:10.1371/journal.pone.0278654)
Supplement: S1 Appendix — (PDF) [file pone.0278654.s002.pdf]

Appendix

Table A1. Variable definitions.

Variable	Definition
IndLoan	The ratio of bank's loans on the industry in which the large private shareholder is located to bank's total loans.
LPS	The shareholding of large private shareholders of city commercial banks.
IP	A dummy variable that equals 1 if the industries that are supported by the industrial policies include large private shareholders.
Size (CNY)	The natural logarithm of the bank's total assets.
LDR	The loan-to-deposit ratio.
Fore	A dummy variable that equals 1 if the bank's shares held by foreign investors.
CAR	The capital adequacy ratio.
Sta	A dummy variable that equals 1 if the state-owned shareholder whose shareholding is higher than the large private shareholder of the bank is peer (in the same industry) of the large private shareholder.
GDPr	The economic growth rate.
Deptr	The deposit growth rate.
SOE	The ratio of total state-owned assets to GDP.
GDP_sec	The ratio of the GDP of the secondary industry to the total GDP.
GDP_tert	The ratio of the GDP of the tertiary industry to the total GDP.

Table A2. The ratio of the number of large private shareholders to large state-owned shareholders in various industries.

This table reports the ratio of the number of large private shareholders to large state-owned shareholders of city commercial banks in different industries. The name of the industry is classified according to the first letter of the "Guidelines for the Classification of Listed Companies Industry" issued by the China Securities Regulatory Commission.

The name of the industry in which the large private (state-owned) shareholder of the city commercial bank is located	The ratio of the number of large private shareholders to large state-owned shareholders
Wholesale and retail	9.50
Real estate	8.00
Leasing and business services	4.56
Manufacturing	2.10
Construction industry	0.51
Financial industry	0.30
Information transmission, software and information technology services	0.30
Mining industry	0.29
Transportation, warehousing and postal industry	0.22
Accommodation and catering	0.08
Electricity, heat, gas and water production and supply industry	0.06
Agriculture, forestry, animal husbandry and fishery	0.00
Culture, sports and entertainment industry	0.00
Water, environment and utilities management	0.00
