# Supplementary material for: Large private shareholders, industrial policies and industrial loans of city commercial banks: Evidence from China
Source: PLoS One. 2022 Dec 8;17(12):e0278654. doi: 10.1371/journal.pone.0278654 (PMC9731413; doi:10.1371/journal.pone.0278654)
Supplement: Attachment — Submitted filename: Response to Reviewers.pdf [file pone.0278654.s003.pdf]

Response to reviewer #1 comments

Dear Reviewer Rayenda Khresna Brahmana,

We really appreciate you for your carefulness and conscientiousness. Your suggestions are really valuable and helpful for revising and improving our paper. According to your suggestions, we have made the following revisions on this manuscript:

1. First of all, kindly note that introduction section plays important role to attract the attention. When the introduction is bad, most likely the reviewers will reject the paper. Unfortunately, the introduction is the weakest part of this paper.

As earlier mentioned, it is confusing. These are my suggestions:
Rather than starting with discussion about china, focus first on what kind of contribution this paper offers to the body of knowledge. Move the first paragraph of introduction to section 2 (literature review) as a new sub-section. Name it as 2.1 Ownership Expropriation and Industrial Loan in Chinese City Commercial Banks.

As I believe the selling point of the paper is the moderating variable, it is important to show the mixed findings between large shareholders and industry loan. Therefore, paragraph 2 of Introduction can be the first paragraph. However, discuss first the mixed findings between large shareholders and industry loan. The author can break it into two paragraphs, where first paragraph discusses the positive relationship, and second paragraph discusses about negative relationship.

Following that paragraph, the author can offer industry policy as the moderating variable. Elaborate also which theory supports the argument that industry policy can strengthen the relationship. It is your second or third paragraph.

Then, show why Chinese city commercial banks are interesting research setting. Why the case of Chinese city commercial banks is important for body of knowledge? How it suits the research context? How researchers from other countries can learn from the case of Chinese city commercial banks? Yes, the author can elaborate it with the existing paragraph 3.

Then follow with the contributions, which is already in the paper (paragraph 4 to 7).

Response 1: Thank you very much for your advice. I have rewritten the Introduction based on your suggestions.

We focus first on what kind of contribution this paper offers to the body of knowledge (paragraph 1).

Indeed, the selling point of the paper is the moderating variable.Therefore, we focus on the moderating effects of industrial policy (paragraph 2). We also elaborate which theory supports the argument that industry policy can strengthen the relationship.

Then, we show why Chinese city commercial banks are interesting research setting (paragraph 3).

2. Meanwhile, section 2 (the literature review) can be divided into three section. Section 2.1 is Ownership Expropriation and Industrial Loan in Chinese City Commercial Banks. Section 2.2 is Theoretical analysis (only discuss the theoretical framework that support the research framework). Section 2.3 is for Hypothesis development. Don't mix theoretical analysis and hypothesis development.

I still don't get which theory that used and tested. It is unclear. This is why it is important to have Section 2.2

Response 2： Thank you for your constructive comments. We have revised the section 2 according to your comments.

Section 2.1 is Ownership Expropriation and Industrial Loan in Chinese City Commercial Banks.

Section 2.2 is Theoretical analysis. We formulate the theory of information asymmetry. This specifically includes adverse selection and moral hazard.

Section 2.3 is for Hypothesis development.

3. The Data and Methodology section is also unclear. Keep it simple and straightforward. 3.1 is for Data, 3.2 Model Specification, and 3.3 is Measures. The 3.2 can provide only the final model for brevity reason.

Add 1 table for variable definition. You can add it in 3.3 or in Appendix. Then, summarize the variable definition in Page 6 t0 7. Keep it simple.

Response 3： We have simplified the Data and Methodology section. And added Appendix TableA1 for variable definition.

4. The results are exhausting and confusing. It obfuscates the main idea of the research. The authors mix and scramble all the tables. My suggestions:

Provide only the main results for hypothesis testing, which are: Table 2 Column 3 and Table 13. Just combine the Table 2 Column 3 and Table 13 as one table. Called it as 4.2 Results

Start with 4.1 descriptive statistics (Table 1) and add correlation matrix.

Add one section and called it 4.3 Robustness check. Then provide Table 4, 16, 17, 18, and 19.

Move Table 3 to section 2.1

Move Table 6 to Section 3.1 or Appendix

Delete the rest of tables.

Add one analysis for robustness check, which is Difference-in-Difference (but this is optional as your results already massive).

Add the moderation plot based on Table 13. Check Dawson (2014).

Response 4: Thank you for your positive comments.
We added the correlation matrix as shown in Table 3 (The Descriptive statistics section).

We provide only the main results for hypothesis testing as shown in Table 4 in 4.2 Results. And add the moderation plot (Fig.3) based on Table 4. Check Dawson (2014).

We moved Table 3 and Table 6 of the original manuscript to section 2.1 and Appendix TableA2, respectively.

In 4.3 Robustness check, we have retained Tables 16, 17, 18, and 19 from the original manuscript, which are now Tables 5, 6, 7, and 8, respectively. We did not retain Table 4 in the original manuscript, which reports the effect of China's banking regulatory policy on the impact of private large shareholders, because it is not relevant to the moderating effect of industrial policy in the Robustness test. This makes the article clearer as you emphasized.

We deleted the rest of tables of the original manuscript.

For DID, this method is often used for policy evaluation. It requires uninterrupted implementation of the policy. But the industrial policies of Chinese local governments do not meet the conditions of DID. Industrial policy is not continuous. For example, an industry that is supported in the current "Five-Year Plan" may not be supported in the next "Five-Year Plan". However, we group the sample according to the criteria of whether the private large shareholders are supported by industrial policies. And test the impact of private large shareholders on banks' industrial loans in two groups. These results as shown in Table 9. This makes our robustness tests more richer and reliable.

5. Add contribution for body of knowledge in Conclusion section.

Add limitation and suggestion for future research in conclusion section.

Response 5: Thank you very much for your valuable comments. We have added contribution for body of knowledge and suggestions for future research in conclusion section. In addition, we have removed the inspirations in Conclusion section (the last two paragraphs of Conclusion section in the original manuscript).

6. For sure, the manuscript is too confusing.

Response 6: We removed irrelevant content and tables and rewrote the main content

based on your suggestion. In addition, we have the help of language editing to ensure that our manuscripts are presented in an intelligible fashion and written in standard English.
