# Supplementary material for: Large private shareholders, industrial policies and industrial loans of city commercial banks: Evidence from China
Source: PLoS One. 2022 Dec 8;17(12):e0278654. doi: 10.1371/journal.pone.0278654 (PMC9731413; doi:10.1371/journal.pone.0278654)
Supplement: Attachment — Submitted filename: Response to Reviewers.pdf [file pone.0278654.s004.pdf]

Response to Academic Editor

Dear Editor LeThanh Ha,

We really appreciate you for your carefulness and conscientiousness. Your suggestions are really valuable and helpful for revising and improving our paper. According to your suggestions, we have made the following revisions on this manuscript:

1.The current version is too long by conducting many robustness tests but unintentionally.
Please review and make the paper more concise.
Authors also should highlight more their contributions and theoretical framework.

Response 1:
We edited the article again to make it more concise. For example, control variables are no longer included in the table of regression results (except Table 4). We have also deleted Table 8 in the original manuscript because its role is similar to Table 7.

We have added a paragraph in the introduction (Lines 86-90), which describes more our contributions and enrichment of information asymmetry theory.

Response to reviewer #1

Dear Reviewer Rayenda Khresna Brahmana,

Thank you again for your recognition of our article. Your suggestion is very helpful to improve the quality of our paper. According to your suggestion, we have made the following modifications to this manuscript:

1.I still do not satisfy with the introduction, specifically, the first paragraph. As per earlier comments: it is important to show the mixed findings between large shareholders and industry loan. One good example is the paper from Graffin et al (2020). Look at their first and second paragraph. Benchmark the way they write the mixed findings. It is not necessarily to have theoretical gap like Graffin et al (2020), you always can show the empirical gap about the mixed findings between the X (independent variable) and Y (dependent variable).

Response 1:

Your comments are very professional and important. At present, there are not many scholars studying the relationship between large shareholders and industrial loans of banks. This leads us to be unable to effectively summarize the mixed relationship. Fortunately, the relationship between large shareholders and the total scale of bank loans has received some attention. Therefore, we show the mixed findings between them.

2.Related to hypothesis development, you need to have three hypotheses. Refer to Brambor et al (2006), Balli & Sorensen (2013) and Dawson (2014) for the important to have three hypotheses in the moderating effect study. The first hypothesis is about the relationship between X and Y. The second hypothesis is about the relationship between M and Y. The last hypothesis is about the interaction term. It is up to your research group, whether want to have separate sub-section for second and third hypothesis.

The theoretical statement for moderating effect is either:

(1)"The positive relationship between X and Y would be strengthened by the moderating effect of M"

(2) "The moderating effect of M would strengthen the positive relationship between X and Y"

Or, (3) you can do several modifications like what Graffin et al (2020).

Response 2:

We now have three hypotheses in the manuscript based on your suggestion.

3.If you do not want to do DID approach, you can argue it in the manuscript, and put it as limitation of study in the conclusion.

Response 3:

We explained the reason for not using DID in the manuscript, and took it as our limitation in the conclusion.

4. I thought that your earlier version has implication for policymakers and industry, but why I can read it again in this revised version? Add it.

Response 4:

We added the inspiration of the paper, which highlights implication for policymakers and industry.

5.I know that PLOSONE will make the final copy-editing, but check again the structure and grammatical errors. For example, "private large shareholders ", "This significantly differs from" (line 41), "differing from" (Line 57), and many more.

Response 5:

We have dealt with grammar errors again by consulting professionals and our school's English teachers.
